# Savings in acute care costs if all older adults treated for fall-related injuries completed matter of balance

**DOI:** 10.1186/s40621-015-0058-z

**Published:** 2015-10-05

**Authors:** Jonathan Howland, Kalpana Narayan Shankar, Elizabeth W. Peterson, Alyssa A. Taylor

**Affiliations:** 1 Department of Emergency Medicine, Boston University Medical Center and Boston University School of Medicine, One Boston Medical Center Place, Dowling 1 South, Boston, MA 02118 USA; 2 Boston Medical Center Injury Prevention Center, Boston, MA 02118 USA; 3 Department of Occupational Therapy, University of Illinois at Chicago, Boston, MA 02118 USA

**Keywords:** Falls, Older adults, Interventions, Community-based, Cost-effectiveness, Emergency departments, Matter of Balance, Massachusetts

## Abstract

**Background:**

Falls among older adults are a common and serious public health problem. Evidence-based fall prevention programs delivered in community settings and targeting older adults living independently are increasingly deployed throughout the nation. These programs tend to be offered by public and private organizations that serve older adults, and recruitment usually occurs through direct marketing to the target population, rather than through referrals from healthcare providers. *Matter of Balance*, a program developed to reduce fear of falling and associated activity restriction in community-dwelling older adults, is currently being delivered in 38 of the 50 United States. In this study, we estimate the one-year medical care cost savings if older adults treated at Massachusetts hospitals for fall-related injuries were referred by healthcare providers to participate in *Matter of Balance*.

**Methods:**

Data from several sources were used for this study. We estimated annual cost savings in older adult falls recidivism for a hypothetical 100 patients presenting at an emergency department for a fall-related injury, assuming that all were referred to, and 50 % completed, *Matter of Balance*. This cost-saving estimate was subsequently expanded based on the actual number (43,931) of older adult patients presenting at, and discharged from Massachusetts emergency departments for all fall-related injuries in 2012. Cost savings were calculated for two additional participation rates: 25 % and 75 %. The return on investment (ROI), was calculated based on the percentage of return per each dollar invested.

**Results:**

The calculated ROI for *Matter of Balance* was 144 %. Statewide savings ranged from $2.79 million assuming a 25 % participation rate to $8.37 million, assuming a 75 % participation rate.

**Conclusions:**

Referral to evidence-based falls prevention programs of older adult patients presenting at EDs with a fall-related injury could reduce subsequent falls and associated treatment costs.

## Background

Falls among older adults are a common and serious public health problem that can cause debilitating, sometimes fatal, injuries and affect subsequent psychosocial status and quality of life. Each year, a third of those 65 years of age or older fall and among this age group, falls are the leading cause of fatal and non-fatal injuries (Centers for Disease Control and Prevention, 2015). In 2013, 2.5 million older adults were treated in emergency departments for non-fatal fall-related injuries and more than 734,000 of these patients were hospitalized. In that year, the direct medical costs for older adult falls was $34 billion, adjusted for inflation (Centers for Disease Control and Prevention, 2015). Even when falls do not require medical attention, the experience can result in fear of falling, which can be psychologically disabling and lead to future falls through physical deconditioning (Howland et al., 1998; Friedman et al., 2002; Delbaere et al., 2004).

Several decades of research on fall prevention have yielded relatively low cost, low-tech interventions that are evidence-based for falls prevention and target older adults living in the community (Gillespie, et al., 2012; Chang et al. 2004). These programs have been classified as *multifactorial*, *multiple*, or *single* (Gillespie, et al., 2012). *Multifactorial* programs consist of falls risk assessment performed by healthcare providers followed by a combination of specific interventions (e.g., review and adjustment of medications that might increase falls risk) designed to address the individual risks for a given patient. *Multifactorial* interventions are typically managed by a primary care physician in a clinical setting. *Multiple* interventions consist of a fixed combination (e.g. cognitive restructuring, exercise, home safety assessment) usually delivered in a community-based group venue, with all participants receiving the same content, regardless of their individual risk factors. These programs often include components designed to increase self-efficacy for fall prevention. For older adults who are too frail to attend group programs in the community, some *multiple* interventions, such as the Otago Exercise Program are designed to be delivered at home by a healthcare provider. Examples of *multiple* programs are Matter of Balance (MOB) (Tennstedt. et al., 1998; Zijlstra et al., 2009), MOB/VLL, the lay-led version of MOB (Healy et al., 2008), and Stepping On (Clemson et al. 2004). *Single* programs consist of one intervention strategy only, such as exercise and/or balance training. These programs are also often delivered to a group, without individualized content. Examples are various versions of Tai Chi (Wolf, et al. 1996; Li et al. 2005) that have been shown to be effective for falls prevention and other exercise programs.

It important to study the cost savings potential of these fall prevention programs because they are increasingly deployed throughout the nation. The community-based non-clinical programs have traditionally been offered by public and private organizations that serve older adults (e.g., Councils on Aging) and recruitment usually occurs through direct marketing to the target population, rather than through referrals from healthcare providers. Nonetheless, it is likely that falls prevention programs will eventually be integrated into the healthcare system as physicians become more engaged in fall risk assessment for their older patients. The CDC has developed the STEADI (Stopping Elderly Accidents, Deaths & Injuries) toolkit, an algorithm for guiding clinical assessment and intervention for fall risk in older adult patients. Given its emphasis on efficient healthcare models that tie improved patient outcomes to payment (Fisher & Friesema, 2013), the Affordable Care Act may facilitate fall risk assessment and referral to community-based falls prevention programs.

Several recent studies have reported on the cost saving potential of both community- and clinic-based falls prevention programs. Based on data from a series of randomized control trials, Carande-Kulis et al. (2014) calculated the net benefit and return on investment (ROI) of three falls prevention programs: the *Otago Exercise Program* (Robertson et al. 2002), *Tai Chi: Moving for Better Balance* (Li, et al., 2005) and *Stepping On* (Clemson, et al., 2004). The *Otago Exercise Program,* a 6-month, individually tailored program delivered in the home by a physical therapist or other healthcare provider and targeting frail older adults, had a one-year net benefit of $121.85 and a ROI of 36 % for each dollar invested. *Tai Chi: Moving for Better Balance*, a 26-week group program targeting community-dwelling older adults, had a one-year net benefit of $529.86 and a ROI of 509 % for each dollar invested. *Stepping On*, a 7-week program that aims to improve falls self-efficacy, encourage behavioral change, and reduce falls by combining community-based group sessions with follow-up home visits by a healthcare provider, had a 14-month net benefit of $134.37 and a ROI of 64 % for each dollar invested (Carande-Kulis et al. 2014).

Wu et al. (2010) conducted a cost-effectiveness analysis of a proposed clinical falls prevention program, the Falls Rehabilitation Program (FRP), which would provide Medicare reimbursement for comprehensive falls risk assessment and remediation for older adults who have fallen at least once in the preceding year. Estimated reduction in falls was calculated at 18 %. These investigators concluded that the national net medical cost savings to all payers for treatment of recurrent falls would be $794 million.

The present study focuses on the cost savings potential of MOB, which, along with its lay-led version, MOB/VLL, has also been the focus of several cost benefit analyses. MOB, as originally developed at Boston University, is an eight-session, cognitive behavioral program, led by healthcare providers (Tennstedt et al., 1998). The program was developed to reduce fear of falling and associated activity restriction in community-dwelling older adults (Tennstedt, et al., 1998). MaineHealth subsequently developed a lay-led version of the program (MOB/VLL), with attention to fidelity to the original version (Healy et al., 2008). MOB is now a licensed program available through MaineHealth’s Partnership for Healthy Aging (MaineHealth).

We selected MOB as the focus for this analysis for several reasons. It is the most widely disseminated falls intervention program in Massachusetts (Howland et al. 2015) and the United States (Centers for Medicare and Medicaid Services 2013). It has been delivered in over 38 states and a conservative estimate is that the 60,000 older adults in the U.S. have participated in the program. Two randomized trials have demonstrated the effectiveness of MOB for enhancing older adults’ falls self-efficacy, which is a measure of fear of falling (Yardley et al., 2005), and increasing activity and perceptions of control over risk for falling (Tennstedt et al., 1998; Zijlstra et al., 2009). The results of one of these trials which was conducted in the Netherlands by Zijlstra et al. (2009) showed a significant difference between intervention vs. control groups on the percent of participants who experienced repeat falls at 14 month follow-up.

With data from the Zijlstra et al. (2009) study, van Haastregt et al. (2013) conducted a cost-effectiveness analysis to determine whether MOB also had effects on healthcare costs. Healthcare costs included direct medical care expenses as well as patient and family costs for professional domestic help, home environmental adaptations, and assistive devices. Healthcare utilization was measured by follow-up phone interviews; costs were calculated using average Netherland costs for specific services. Controls received routine care. At 14-month follow-up, the only significant difference in mean group cost was for physiotherapy. This study did not stratify by falls risk nor by those who had recently suffered a fall or falls-related injury.

The Centers for Medicare and Medicaid Services (Centers for Medicare and Medicaid Services (2013)) conducted a retrospective cohort study evaluating MOB. Compared to matched controls, older adults who had participated in the MOB program (likely mostly MOB/VLL programs) had, during the post-participation year, significantly lower health care costs for all Medicare reimbursements (Centers for Medicare and Medicaid Services 2013).

Miller et al. (2011) conducted a cost analysis on the lay version (MOB/VLL) of MOB using data from programs they had evaluated in Texas, as well as other sources. They concluded that MOB/VLL would yield a positive ROI as a consequence of averted healthcare costs if 7 falls were prevented among a 140 participating older adults (≥50 years of age), assuming that 100 completed the program.

In the present paper, we use data from several sources to estimate the one-year medical care cost savings if older adults treated at Massachusetts hospitals for fall-related injuries were referred by healthcare providers to participate in MOB/VLL. We focus on ED patients treated for fall injuries because: (1) older adults experiencing a fall are at increased risk for a subsequent fall and (2) the fall event may constitute a “teachable moment” wherein older adults may be most motivated to participate in fall prevention activities recommended by a healthcare provider. We focus on MOB/VLL because: (1) there is evidence that MOB reduces repeat falls that require medical attention (Zijlstra et al., 2009) and (2) it is widely disseminated in the US (Centers for Medicare and Medicaid Services 2013).

Our estimates of reductions in older adult repeat falls requiring medical attention derives from a randomized trial of MOB because comparable data are not available for MOB/VLL. Our program cost estimates come from an analysis of MOB/LLV because this is the most widely deployed of the two versions. (MOB and MOB/VLL are essentially the same except for the credentials of program leaders.) In Massachusetts, and likely elsewhere, the distinction between the versions is blurred because some MOB/VLL programs are in fact led by volunteer healthcare professionals (Howland et al., 2015).

To our knowledge, this is the first study to estimate the potential of MOB/VLL for reducing costs related to repeat emergency department (ED) visits and inpatient care for treatment for older adults’ fall-related injuries.

## Methods

The aim of this analysis was to estimate the ROI if community-dwelling older adults treated at EDs for injurious falls were advised by attending healthcare providers to participate in MOB/VLL. Our outcome was averted payments by third-party insurers for the treatment of a repeat injurious fall within a year of the index fall. These costs included the price paid for ED and inpatient care, but not other fall-related costs, such rehabilitation care or assistive devices. Estimates of the effectiveness of MOB/VLL in reducing repeat falls requiring medical care were derived from the literature, as were estimates for other critical variables, such as the cost of the intervention and the rate of repeat injurious falls among older adults. We could find no published studies with which to estimate the participation rate of older adults referred by healthcare providers to community-based falls prevention programs. Accordingly, this variable was treated as a parameter for which calculations were repeated for various values. ROI was calculated pursuant to standard economic analysis methods (Carande-Kulis et al. 2014; Miller et al., 2011). Our approach and findings were reviewed by two economists, one with expertise in injury control and the other with exptertise in health-related behavioral economics. Details on the derivation of variable estimates follow.

### ED recidivism for older adult fall-related injuries

To estimate the proportion of older adults treated in EDs for fall-related injuries who return within one year with a subsequent fall-related injury (fall recidivism), we used data from a study conducted by Russell et al. (2010). This study prospectively followed 698 patients for one year after treatment for a fall or fall-related injury (Russell et al. 2010). Within one year of discharge for the initial fall injury, approximately 18 % (126/698) returned to the ED for a fall-related visit.

### Proportion of older adult fall-injured patients admitted to hospital

To estimate the proportion of older adult patients with fall-related injuries who are discharged from the ED vs. admitted to hospital, we used 2012 data from a Massachusetts Department of Public Health (MDPH) report on statewide unintentional older adult (≥65 years) falls (Hackman 2015). Of 65,529 falls statewide that required hospital-based medical attention that did not result in immediate death, 33 % (21,598) were admitted as inpatients and 67 % (43,931) were discharged.

### Cost of ED visits and inpatient care for older adult fall-related injuries

To estimate the mean cost of an ED visit for an older adult fall-related injury in Massachusetts in 2013, we used data compiled by the aforementioned MDPH report (Hackman, 2015). We divided the total cost for older adult fall-related ED visits by the number of ED admissions for falls that were discharged without hospitalization ($124 M/43931 = $2,823).

To estimate the mean cost of a hospitalization for an older adult fall-related injury in Massachusetts in 2013, we used data compiled by the same MDPH report (Hackman, 2015). We divided the total cost for older adult fall related hospitalization by the number of inpatient admissions for falls ($550 M/24,465 = $25,465).

### MOB/VLL impacts on repeat falls

To estimate the impact of MOB/VLL in reducing the likelihood of repeat falls requiring a return to hospital, we used findings from a randomized trial of MOB that was conducted in the Netherlands and that measured the effects of MOB on repeat fallers at 14 months follow-up (Zijlstra et al. 2009). This study found a 22 % decrease (intervention vs. control groups) in the percent of participants experiencing repeat falls that required an ED visit at 14 months follow-up.

### MOB/VLL costs

To estimate the per capita costs of intervention, we used data from a cost analysis of the implementation of MOB/VLL in South Florida (Page et al. 2012). These investigators estimated that during the second year of implementation the average cost for an individual completing MOB/VLL was $176. Miller et al. (2011) calculated the costs of MOB/VLL as $189 per program completer. This calculation included startup costs (e.g., leader training). We chose to use costs during the second year of implementation, rather than include startup costs because we considered the former to more relevant, assuming institutionalization of referrals to MOB/VLL. Nonetheless, the difference between the costs estimates of Page et al. (2012) and Miller et al. (2011) was small.

### Referral rate

Because it is not possible to predict which patients will return to hospital for a fall-related injury, our estimates assume that all older adult fall-injured patients should be prophylactically referred to the MOB/VLL program.

### Patient participation rate in falls prevention programs

Because most all older adults who have thus far participated in MOB or MOB/VLL have elected to do so without a referral or advice from their physician, there are no data on the possible uptake rate for patients should community falls prevention become an integral part of medical care for older adults at high risk for falling. Therefore, this parameter was designated ʎ and cost savings were calculated for three values: 25 %, 50 % and 75 %.

### Data analysis

First, annual cost savings were estimated for 100 hypothetical older adult patients presenting at an ED for a fall-related injury, assuming that all were referred to, and 50 % completed, MOB/VLL. Next, this cost-saving estimate was scaled up based on the actual number (43,931) of older adult patients presenting at, and discharged from Massachusetts EDs for all fall-related injuries in 2012. By way of sensitivity analysis, these calculations were repeated for two other values (25 % and 75 %) of ʎ. ROI was calculated based on the percentage of return per each dollar invested ([net total cost savings/cost of program] × 100) (Carande-Kulis et al. 2014; Miller et al., 2011).

## Results

Assuming a hypothetical sample of 100 older adults discharged from Massachusetts EDs for fall-related injuries, 18 (18 %) will revisit an ED for a subsequent fall-related injury within one year. Of these, 12 (67 %) will be discharged from the ED and 6 (33 %) will be hospitalized. The cost of ED treatment will be $33,876 (12 × $2,823) and the cost of inpatient care will be $152,790 (6 × $25,465), for a total cost of $186,666 ($33,876 + $152,790) (Table 1).

**Table 1:** Cost Savings if 50 % of Referred Patients Completed MOB

1-Year Savings if All Fall Injured Older Adults were Referred to MOB and 50 % Participated	CONDITION		
	NO MOB	100 % Referral/ 50 % Adherence	
		50 % WO/MOB	50 % W/MOB
	*N* = 100	*N* = 50	*N* = 50
Return 1 Year	18	9	7 ([1-.22] × 9)
Discharged from ED	12	6	4.7
Admitted	6	3	2.3
ED Costs @ $2823	$33876	$16938	$13268
	(12 × $2823)	(9 × 2823)	(4.7 × 2823)
Hosp. Costs @ $25465	$152790	$76395	$58570
	(6 × $25465)	(3 × $25465	(7 × .33 × $25465)
Sub Total	$186666	$93333	$71838
Cost of MOB	$0	$0	$8800 (176 × 50)
			$80638
Total	$186666	$173971 ($93333 + $80638)	
Net Savings	$12695 ($186666 - $173971)		
Savings Scaled to state	$5.58 Million ([43931/100] × $12695)		
ROI	144 % ([12695/8800] × 100)		

Assuming that all 100 patients were referred to MOB/VLL and that 50 % opted to participate and complete the program, within a year, there will be an estimated 9 (.18 × 50) individuals requiring medical attention among non-participants and 7 ([1-.22] × [.18 × 50]) individuals among MOB/VLL completers who have a repeat fall requiring medical attention. Among the non-participants, 6 (9 × .67) will be discharged from the ED, at a cost of $16,938 (6 × $2,823) and 3 (9 × .33) will be hospitalized, at a cost of $76,395 (3 × $25,465). Thus, the total cost for treatment of the non-participating patients will be $93,333 ($16,938 + $76,395) (Table 1).

Among the patients participating in MOB/VLL, 4.7 (7 × .67) will be discharged from the ED, at a cost of $13,268 (4.7 × $2,823) and 2.3 (7 × .33) will be hospitalized at a cost of $58,570 (2.3 × $25,465), for a total medical care cost of $71,838. To this we added $8,800 ($176 × 50), the cost of providing 50 patients with MOB/VLL classes), resulting in a total treatment cost for the 50 participating patients of $80,638 ($71,838 + $8,800). Therefore, the total cost of treating the 100 patients, assuming an 18 % falls recidivism rate and a 50 % participation rate in MOB/VLL, will be $173,971 ($93,333 + $80,638). The difference in costs between no patients referred to, or completing, MOB/VLL and 100 % referral rate, with 50 % completing the program, is $12,695 ($186,666 - $173,971) (Table 1).

In 2012, there were 43,931 older adults treated and discharged from Massachusetts EDs for fall related injuries. Thus, scaled up to a statewide level, the net savings would equal $5.58 million ([43,931/100] × $12,695). If 25 % of patients opted to participate in, and complete MOB/VLL, savings statewide would be $2.79 million; if 75 % opted to participate in, and complete, MOB/VLL savings would be $8.37 million.

At 50 % uptake, the calculated ROI was 144 % ([$12,695/[$176 × 50]] × 100).

## Discussion

This analysis suggests that participation in MOB/VLL could reduce costs for the treatment of repeat fall-related injuries. Given the high cost of falls and fall-related injuries, initiating a policy to refer older adults treated for fall-related injuries to evidence-based falls prevention programs could result in cost savings to healthcare systems. Because we know of no comparable investigation estimating the cost impacts of MOB/VLL, we cannot compare our findings to similar studies.

The evidence used to reach this conclusion was not the result of a prospective study, but rather used data synthesized from several relevant studies. Accordingly, certain limitations should be considered.

First, for our purposes, the evidence that participation in MOB/VLL will reduce falls recidivism is limited in several respects. The metric used by Zijlstra et al. (2009) was the number of repeat fallers, rather than number of falls, requiring medical attention. We have used number of fallers as a surrogate for number of falls. It is likely, therefore, that our cost saving estimates are underestimated, because during the follow-up period, some fallers may have experienced multiple falls requiring medical attention. It should also be noted that MOB was not originally designed to impact falls, but rather falls self-efficacy and activity avoidance due to fear of falling. Neither the original randomized trial of MOB (Tennstedt et al., 1998) nor the Zijlstra et al. (2009) study found effects for falls per se. Several published studies (Healy et al., 2008; Smith et al. 2014) have reported pre- and posttest fall reductions among subjects participating in MOB/VLL, but these studies have used uncontrolled designs and thus their internal validity is questionable. On the other hand, the two randomized trials of MOB have found strong evidence that MOB increases falls self-efficacy, sense of control over the management of falls risk, and increased activity levels. These outcomes are consistent with effects for falls risk reduction. Zijlstra et al. (2009) suggest that a reason why MOB might affect repeat falls, but not all falls, is because a one-time fall may involve an element of chance while a repeat fall more likely reflects an inherent risk. Thus, the metric that counts all falls includes a certain amount of random error, while a metric that counts repeat falls may be more precisely assessing changes in risk.

Several other characteristics of the Zijlstra et al. (2009) study should be noted. As in the original version, the MOB program in the Netherlands study was led by healthcare professionals. Since the content of the original and lay led versions are virtually the same, it is reasonable to assume that the Zijlstra et al. (2009) findings generalize to MOB/VLL, the version of MOB most widely distributed in the US. Nonetheless, no study has directly compared the two versions of MOB. The version of MOB studied by Zijlstra et al. (2009) included a booster session that is not included in the versions typically conducted in the US. It is possible that the intervention effect for repeat fallers was enhanced by this booster. Also, the Zijlstra et al. (2009) study enrolled older adults ≥ 70 years of age, whereas the present analysis is based on those ≥ 65 years. This age difference might affect our findings, but it is difficult to predict the direction of effect.

Second, the strength of our estimate on falls recidivism is that it involved a large number of patients (Russell et al. 2010). However, the recidivism rate was calculated by patient report and by medical records at the hospital at which the patients had initially presented with a fall injury. Some patients may have sought care for a repeat fall at another hospital, were transported to another hospital by emergency medical service, or were unable to remember visiting an ED for a fall. Thus, the rate of recidivism may be underestimated, in which case our estimates of cost savings would be higher.

Third, the cost savings estimates we calculated were limited to ED and inpatient care for repeat fall injuries. We did not include costs associated with follow-up rehabilitation, stays in long term care facilities, home health services, and other costs that may be incurred as a consequence of an injurious fall. Thus, our findings likely underestimate the total cost savings resulting from reduction in repeat falls. Also, it is possible that the effects of MOB or MOB/VLL may extend beyond the one-year follow-up periods typical of most studies. Our analysis was limited to estimating one-year cost savings, but these saving may be repeated for post-intervention multiple years. Moreover, in the retrospective cohort study conducted by Centers for Medicare and Medicaid Services (2013)), MOB or MOB/VLL participants matched with non-participants had significantly reduced overall healthcare costs ($938) and reduced mortality in the year after completing of the program (2.4 % of MOB or MOB/VLL participants died during the post-program year vs. 4.2 % of controls). The cost reductions appeared unrelated to costs associated with treatment for falls, but investigators did not examine repeat falls per se. It is not unreasonable to hypothesize that an increase in falls self-efficacy could generalize to other areas of health. Therefore, MOB or MOB/VLL may reduce overall healthcare costs beyond our estimates of the saving for treatment of repeat fall injuries.

In the absence of data on program uptake among patients referred by their physician, we calculated cost savings for three values of this parameter. Wu et al. (2010) noted that in clinical trials of falls prevention interventions, 45 %-85 % of invited older adults participated. In their cost effectiveness analysis of the FRP program, these investigators estimate a 50 % participation rate based on clinical trial participation. Because patients who participate in clinical trials may be different from the general population, we chose to treat this variable as a parameter and calculate cost savings for three values.

The efficacy of several community-based programs in reducing risk and incidence of falls is well established, at least for follow-up periods of a year or so. Demonstrating the cost savings potential of these programs is the next step towards ensuring that these programs become an integral part of healthcare for older adults. The results of this exercise in cost analysis are not definitive; further study is required to determine if the finding by Zijlstra et al. (2009) are replicable and generalizable to older adult populations in other countries. Better data is needed to estimate the uptake of community falls prevention programs when patients are referred by their primary care physicians or referred on discharge from an emergency department or inpatient hospitalization.

Other falls prevention programs have been shown to be cost effective in reducing falls (Carande-Kulis et al. 2014) and the aforementioned Centers for Medicare and Medicaid Services (2013) study indicates that MOB/VLL reduces total health care costs during the post participation year. Indeed, the ROI we calculated exceeds that calculated by Carande-Kulis et al. (2014) for *Otago* and *Stepping On*. Thus, while further research could increase the precision of our estimates, it is not unreasonable to conclude that MOB or MOB/VLL could contribute to reduction in older adults’ repeat falls and associated costs.

## Conclusions

Referral to evidence-based falls prevention programs of older adult patients presenting at EDs with a fall-related injury could reduce subsequent falls and associated treatment costs.

## Abbreviations

ROI: Return on investment
MOB: Matter of balance
MOB/VLL: Matter of balance lay-led version
CDC: Centers for Disease Control and Prevention
STEADI: Stopping elderly accidents, deaths and injuries
CMMS: Centers for Medicare and Medicaid Services
MDPH: Massachusetts Department of Public Health
FRP: Falls Rehabilitation Program
